# Medical care or clean energy? The impact of medical insurance on rural households’ use of clean cooking energy

**DOI:** 10.3389/fpubh.2026.1870573

**Published:** 2026-06-29

**Authors:** Guanghua Lin, Wenqing Wu, Yan Yang, Licheng Zhang

**Affiliations:** College of Economics and Management, Nanjing Agricultural University, Nanjing, China

**Keywords:** clean energy use, health economics, preventive health investment, rural China, rural medical insurance

## Abstract

**Background:**

Promoting the use of clean cooking energy is crucial for safeguarding the health of rural households. However, despite its role as a health-enhancing good, few studies have examined the relationship between clean energy use and other forms of health investment. While medical insurance improves rural households’ access to medical care, its impact on clean cooking energy use remains unclear.

**Methods:**

Using micro-level household panel data from 2012 to 2022, this study employs a two-way fixed effects model to examine the impact of medical insurance on rural households’ use of clean cooking energy. In addition, we conduct a series of robustness checks to verify the reliability of the main findings. We further perform heterogeneity analyses to examine whether the effect of medical insurance on clean cooking energy use differs by household age structure, household assets, and medical access barrier.

**Results:**

The results show that: (1) medical insurance participation significantly reduces the probability that rural households use clean cooking energy; (2) medical insurance increases the probability of hospitalization, out-of-pocket medical expenditures, inpatient expenditures, total household medical expenditures, and the share of medical spending in the household budget; and (3) the negative effect is more pronounced among households with higher old-age dependency ratios, lower asset holdings, and greater barriers to medical access.

**Conclusion:**

This study reveals an unintended consequence of medical insurance and provides important insights for governments seeking to promote both insurance coverage and clean cooking energy use. This issue is particularly relevant for many developing countries, where health protection and clean energy promotion are often pursued simultaneously under tight household budget constraints.

## 1 Introduction

Rural households around the world have long relied on solid fuels such as firewood and coal for cooking and heating. The combustion of solid fuels releases large quantities of harmful pollutants, including PM2.5, carbon monoxide, and polycyclic aromatic hydrocarbons, thereby increasing the risks of stroke, ischemic heart disease, chronic obstructive pulmonary disease, and lung cancer (1–4). According to the World Health Organization, about 2.1 billion people worldwide still rely on solid fuels for cooking, and household air pollution is responsible for approximately 2.9 million premature deaths each year (5). Compared with solid fuels, clean energy sources such as electricity, liquefied petroleum gas, and natural gas generate substantially fewer air pollutants. Therefore, promoting the use of clean cooking energy is not only an environmental policy issue but also an important public health strategy.

Given the health benefits associated with clean energy use, rural households’ use of clean cooking energy can be viewed as a health investment decision. Following the health capital framework, households allocate limited resources across different goods and services that contribute to the production of health, and this allocation depends on the relative costs and expected returns of alternative inputs (6). Therefore, changes in the costs of other health-related goods and services may influence rural households’ decisions regarding clean cooking energy use. However, existing studies have largely treated clean energy use as an energy or environmental decision, focusing on the impact of energy availability (7–9), environmental regulations (10, 11), and household demographic characteristics (12, 13) on clean energy use. Less attention has been paid to its role as a preventive health investment, and even less is known about how clean energy use interacts with other health-producing inputs, such as medical care. To address this gap, this study analyzes rural households’ clean cooking energy use from a health investment perspective and examines how changes in the cost of medical services affect household clean energy decisions.

China provides a useful setting for examining this question. Since 2003, China has progressively expanded rural medical insurance coverage through the New Rural Cooperative Medical Scheme (NCMS) and, subsequently, the integrated Urban–Rural Resident Basic Medical Insurance (URRBMI). Rural residents enroll in these schemes by paying an annual premium and receive reimbursement for eligible outpatient and inpatient expenditures in accordance with policy rules (14, 15). Reimbursement rates vary across regions, years, and levels of medical institutions. In the early stage of the NCMS, the inpatient reimbursement rate was roughly 40%. With the expansion of the program and the increase in government subsidies, the level of financial protection gradually improved. By 2013, the average reimbursement rates for eligible outpatient and inpatient expenses had exceeded 50 and 75%, respectively, while reimbursement for general outpatient care was about 40% in many areas (16, 17). This institutional change substantially reduced the cost of medical care and provides an opportunity to examine how cheaper access to medical services affects households’ allocation across different health investments.

Medical insurance may affect clean cooking energy use through two related mechanisms. First, as a formal health input, medical care becomes relatively cheaper under insurance coverage, making it more attractive within the household’s health investment portfolio. Due to moral hazard, households may increase their use of medical services and reduce their demand for other health-related investments (18). Existing studies show that medical insurance may weaken individuals’ incentives to engage in health-promoting behaviors, leading to more smoking and drinking and less physical exercise (19–22). Since clean cooking energy is also a health-enhancing good, medical insurance may induce households to substitute medical care for clean cooking energy, thereby reducing the likelihood of using clean cooking energy.

Second, regular health check-ups remain uncommon in rural China. In particular, annual examinations and proactive health management are still inadequate among rural adults aged 60 years and above (23), while undiagnosed and uncontrolled chronic diseases remain prevalent among Chinese rural residents (24). After enrolling in medical insurance, improved access to medical services may make households more likely to detect previously undiagnosed illnesses during healthcare visits, which can further increase medical expenditures (25–27). Some studies have shown that although the New Cooperative Medical Scheme (NCMS) improves household health outcomes (27) and reduces the likelihood of illness (28), it may also increase the likelihood that households’ fall into poverty (29). Given household budget constraints, the increase in medical spending induced by medical insurance participation may crowd out other health-related investments, including the use of clean cooking energy.

Although many studies have examined the health and expenditure outcomes of medical insurance, its impact on clean energy use remains unclear. Against this background, this study employs micro-level household panel data from 2012 to 2022 to examine the impact of medical insurance on rural households’ use of clean cooking energy. We find that participation in medical insurance significantly reduces the probability of using clean cooking energy, and this result remains robust across a range of robustness checks and additional identification analyses. We also find that medical insurance improves access to medical care and shifts household expenditures toward medical services, providing evidence for the proposed health investment substitution mechanism.

This study makes three contributions. First, it extends the literature on clean cooking energy use by conceptualizing clean energy as a preventive health investment rather than merely an energy-consumption choice. This perspective highlights that household clean energy decisions depend on the relative costs of other health-producing inputs. Second, this study contributes to the literature on the behavioral consequences of medical insurance by documenting an unintended effect on household energy choices. While prior studies have mainly examined healthcare utilization, financial protection, and health behaviors, we show that medical insurance may also reshape preventive health investments. Third, this study bridges health policy and household clean energy use by providing evidence that medical care and clean energy may act as substitutes in the production of health capital.

The remainder of this paper proceeds as follows. Section 2 develops the research hypotheses. Section 3 describes the data, variables, and model specification. Section 4 reports the empirical results, including the baseline results, robustness checks, endogeneity analyses, heterogeneity tests, and further discussion. Section 5 concludes by summarizing the main findings and outlining their policy implications.

## 2 Research hypotheses

Clean energy use can be understood as a preventive health investment. By reducing household air pollution, clean energy helps lower exposure to harmful pollutants and decreases pollution-related health risks. Within a utility-maximization framework, rural households allocate limited resources across different health-producing inputs, including preventive inputs such as clean energy and curative inputs such as medical care. Accordingly, the decision to use clean energy depends not only on its own cost and health benefits but also on the relative costs and returns of alternative health inputs. When the cost of medical care changes, households may reallocate resources between medical care and clean energy in order to maximize utility (30).

Medical insurance alters this allocation by reducing the cost of medical care and improving access to health services. Compared with uninsured households, insured households face lower outpatient and pharmaceutical expenses. For example, outpatient costs at township health centers may decline by approximately 40 to 75% (16, 17). By reducing the cost of medical care, medical insurance increases its relative attractiveness within the household’s health investment portfolio. From the perspective of substitution among health investments, households may therefore substitute formal medical care for clean energy, reducing their incentive to use clean energy.

In addition to this substitution effect, medical insurance may also generate a budget crowding-out effect. Rural residents in China generally lack the habit of regular health check-ups, and health information is characterized by substantial incompleteness. In 2012, the health examination rate among rural residents was only 27.35% (31). In the absence of insurance coverage, high outpatient costs may lead households to delay seeking care when experiencing mild symptoms such as headaches or fatigue, leaving many chronic conditions undetected (24). As medical insurance lowers the cost of seeking care, insured households are more likely to use medical services and discover latent illnesses during healthcare visits (32–36). This information mechanism may release previously suppressed medical demand and increase household medical expenditures. Under household budget constraints, rising medical spending may crowd out other categories of expenditure, including the use of clean energy (37).

Furthermore, the substitution between medical care and clean energy may be reinforced by the moral hazard effect of insurance. After obtaining insurance coverage, rural households face lower marginal medical costs and may increase their use of medical services. This may weaken their incentive to obtain health benefits through preventive investments, such as clean energy use. Therefore, medical insurance may reduce rural households’ use of clean energy by increasing the relative attractiveness of medical care and shifting household resources toward medical expenditures.

Based on the above discussion, this study proposes the following hypotheses:

*H1*: Participation in medical insurance reduces rural households’ use of clean energy.

*H2*: Participation in medical insurance increases medical care utilization and medical expenditures, thereby crowding out households’ use of clean energy.

## 3 Materials and methods

### 3.1 Data source and sample construction

This study uses data from the China Family Panel Studies (CFPS), a nationally representative longitudinal survey conducted by the Institute of Social Science Survey at Peking University. CFPS collects information at the individual, household, and community levels and covers a wide range of topics, including economic activities, education, family relations, migration, and health. We draw on six waves from 2012 to 2022.

To identify the effect of medical insurance participation on household clean energy use, we construct a balanced rural household panel. We first retain rural households and exclude urban samples. We then keep households whose members primarily reside at home and participate in daily household activities, because energy choice is most directly linked to the behavior and welfare of resident household members. Finally, we remove observations with missing key variables or obvious logical inconsistencies. After cleaning and matching the six survey waves at the family level, the final estimation sample contains 8,780 household-wave observations.

### 3.2 Variables

#### 3.2.1 Dependent variable

The dependent variable is household clean cooking energy use (CleanEnergy). The CFPS questionnaire asks households about their primary cooking fuel, with response options including firewood, coal, gas, natural gas, solar energy, biogas, and electricity. From the perspective of indoor air pollution, we define firewood and coal as traditional energy sources, while all other options are classified as clean energy sources.

CleanEnergy is constructed according to the household’s current primary cooking fuel in each survey year. Specifically, if household *i* mainly uses a clean energy source for cooking in year *t*, CleanEnergy is assigned a value of 1; if household *i* mainly uses firewood or coal for cooking in year *t*, CleanEnergy is assigned a value of 0. Therefore, this variable captures the household’s current clean cooking energy use status in each survey year. It should be noted that, due to data limitations, this measure does not capture cases in which rural households use clean energy and traditional solid fuels simultaneously. Nevertheless, the primary cooking fuel reflects the household’s dominant cooking energy choice and captures the main pattern of household cooking energy use.

#### 3.2.2 Independent variable

The core independent variable is the household medical insurance participation rate (InsRate). To ensure consistency across survey waves, participation is identified using enrollment in the NCMS for 2012–2018 and enrollment in NCMS or URRBMI for 2020–2022. Considering that household energy use decisions are primarily shaped by the health protection, labor supply, and resource allocation decisions of working-age members, we further restrict the calculation of insurance participation to household members aged 16–60. The household participation rate is measured as the proportion of insured individuals among household members aged 16–60. This measure captures both the extent of insurance coverage within the household and differences in household size; a higher value indicates a higher overall level of household insurance participation.

#### 3.2.3 Control variable

We control for household-head, household, and regional characteristics. Household-head controls include gender, age, education, marital status, and Communist Party membership.

Household controls include household size, logged household income, household net assets, and minimum-living-guarantee status (dibao). These variables are included because they may simultaneously affect medical insurance participation and clean energy use. For example, household size may influence both insurance enrollment decisions and energy demand, while household income and assets capture economic capacity, which affects the ability to pay for insurance premiums and use clean energy. Controlling for these factors helps reduce potential omitted-variable bias and improves the identification of the effect of medical insurance on clean energy use.

Households relying on traditional cooking fuels are often also low-income households, and the Chinese government commonly provides medical insurance premium subsidies to especially disadvantaged families. Therefore, we control for minimum-living-guarantee status (dibao) to reduce potential omitted-variable bias arising from household poverty and related policy support, since economically disadvantaged households may be more likely to receive low-income assistance while also facing greater constraints in using clean energy.

At the regional level, we control for logged provincial GDP to account for differences in provincial economic development, which may affect both medical insurance participation and clean energy use through local public service capacity, infrastructure conditions, energy supply, and household purchasing power. Controlling for this variable allows us to more accurately identify the effect of household medical insurance participation on clean energy use.

Table 1 reports the descriptive statistics of the main variables and provides brief definitions of each variable. In addition, comparison with recent CFPS-based studies on rural household cooking energy choices shows that the descriptive statistics of the final sample are broadly comparable to those of existing rural household samples (38, 39).

**Table 1:** Descriptive statistics of the main variables.

Variables	Definition	Sample	Mean	SD	Min	Max
CleanEnergy	Mainly uses clean cooking energy = 1; mainly uses firewood or coal = 0	8,780	0.499	0.500	0.000	1.000
InsRate	Number of insured household members aged 16–60 divided by the total number of household members aged 16–60.	8,780	0.602	0.287	0.000	1.000
Gender_head	Female = 0, Male = 1	8,780	0.592	0.491	0.000	1.000
Age_head	Age of the respondent	8,780	52.067	12.073	16.000	89.000
Edu_head	Years of schooling completed	8,780	6.728	3.997	0.000	19.000
Marry_head	In a marital union = 1, otherwise = 0	8,780	0.909	0.288	0.000	1.000
Party_head	Communist Party member = 1, otherwise = 0	8,780	0.047	0.213	0.000	1.000
Familysize	Total population of the family	8,780	4.073	1.904	1.000	15.000
IncomeFam	Total household income, including off-farm income, agricultural income, property income, and other income	8,780	10.391	1.164	0.000	13.911
Asset	Total household assets minus total household liabilities	8,780	11.875	4.266	−14.327	17.290
Subsidy_low	Receiving subsistence allowance = 1, otherwise = 0	8,780	0.079	0.271	0.000	1.000
ln_gdp	Logged provincial GDP	8,780	10.015	0.748	8.607	11.795

Descriptive statistics are reported for the regression sample used in the baseline estimations.

### 3.3 Empirical strategy

#### 3.3.1 Baseline model

To estimate the impact of medical insurance on household clean energy use, we employ a two-way fixed-effects model with village and year fixed effects:


$${\text{CleanEnergy}}_{\mathrm{it}}=\alpha_{0}+\alpha_{1}{\text{InsRate}}_{\mathrm{it}}+\alpha_{2}X_{\mathrm{it}}+\varphi_{c}+\lambda_{t}+\varepsilon_{\mathrm{it}}$$

Where ${\text{CleanEnergy}}_{\mathrm{it}}$ is the dependent variable, indicating whether household i mainly uses clean cooking energy in year t ; ${\text{InsRate}}_{\mathrm{it}}\;$ is the key independent variable, measured by the medical insurance participation rate of household i in year t ; $X_{\mathrm{it}}$ denotes a set of control variables; $\varphi_{c}$ represents village-level fixed effects, which capture time-invariant unobservable factors at the village level, such as local energy supply conditions and energy infrastructure. Since energy availability is closely related to local characteristics and is largely determined at the village level, including village fixed effects helps mitigate potential confounding from local energy supply conditions; $\lambda_{t}$ denotes year fixed effects, which control for common shocks varying over time; and $\varepsilon_{\mathrm{it}}$ is the random error term.

#### 3.3.2 Mechanism model

To investigate the proposed channels, we further estimate the following mechanism equation:


$${\text{Mech}}_{\mathrm{it}}=\beta_{0}+\beta_{1}{\text{InsRate}}_{\mathrm{it}}+\beta_{2}X_{\mathrm{it}}+\varphi_{c}+\lambda_{t}+\xi_{\mathrm{it}}$$

Where ${\text{Mech}}_{\mathrm{it}}$ denotes the mechanism variable, including hospitalization, out-of-pocket medical expenditure, inpatient expenditure, total household medical expenditure, and the share of medical expenditure in the household budget. ${\text{InsRate}}_{\mathrm{it}}$ is the key independent variable, measured by the medical insurance participation rate of household i in year t ; $X_{\mathrm{it}}$ denotes the control variables; $\varphi_{c}$ represents village-level fixed effects; $\lambda_{t}$ denotes year fixed effects; and $\xi_{\mathrm{it}}$ is the random error term.

## 4 Results

### 4.1 Baseline results

Table 2 reports the baseline estimates. Column (1) includes only village and year fixed effects; column (2) further adds household-head characteristics; and column (3) additionally controls for household and regional characteristics. Across all specifications, the coefficient on InsRate is negative and significant. In the fully controlled specification, the estimated coefficient of InsRate is −0.044 and statistically significant at the 5% level, indicating that a higher household medical insurance participation rate is associated with a lower probability of using clean energy as the primary cooking fuel.

**Table 2:** Baseline regressions.

Variables	(1)	(2)	(3)
	CleanEnergy		
InsRate	−0.063***	−0.032*	−0.044**
	(0.019)	(0.019)	(0.020)
Gender_head		−0.035***	−0.034***
		(0.012)	(0.011)
Age_head		−0.004***	−0.004***
		(0.001)	(0.001)
Edu_head		0.008***	0.007***
		(0.002)	(0.002)
Marry_head		−0.065***	−0.062***
		(0.024)	(0.023)
Party_head		0.036	0.030
		(0.024)	(0.024)
Familysize			−0.019***
			(0.004)
IncomeFam			0.043***
			(0.005)
Asset			−0.001
			(0.001)
Subsidy_low			0.003
			(0.023)
ln_gdp			−0.053
			(0.072)
Constant	0.537***	0.739***	0.914
	(0.013)	(0.042)	(0.727)
Observations	8,780	8,780	8,780
*R*-squared	0.424	0.438	0.446
Village FE	Yes	Yes	Yes
Year FE	Yes	Yes	Yes
Cluster	fid	fid	fid

Cluster-robust standard errors, clustered at the household level (fid), are reported in parentheses. Village and year fixed effects are included in all specifications. ***p < 0.01, **p < 0.05, *p < 0.10.

### 4.2 Robustness checks

We conduct a series of robustness checks, the results of which are reported in Table 3. To mitigate potential reverse causality arising from contemporaneous insurance participation, we replace the current participation rate with its one-period lag. Column (1) shows that the coefficient on L_InsRate remains significantly negative. Next, to address concerns that time-varying local policies and village-level shocks may confound the estimated relationship, we replace the separate village and year fixed effects with village-by-year fixed effects. This specification absorbs unobserved factors that vary at the village-year level, such as changes in local clean energy promotion policies, energy infrastructure, medical resource allocation, local subsidy programs, and other evolving socioeconomic conditions. As shown in Column (2), the coefficient on InsRate remains significantly negative, suggesting that the baseline finding is not driven by time-varying village-level policy shocks or local implementation differences. In addition, the number of children may constitute an important omitted variable, as it can affect household medical insurance participation while also influencing clean energy use through household dependency burdens, expenditure structure, and cooking demand. We therefore add the number of children as an additional control. Column (3) indicates that the coefficient of InsRate remains negative and significant. Furthermore, households that moved across villages during the sample period may have experienced changes in housing conditions, kitchen facilities, and cooking equipment, and such residential changes may themselves directly affect household energy choices, thereby confounding the identification of the net effect of medical insurance. To address this concern, we exclude households that relocated across villages during the sample period. Column (4) shows that the core coefficient remains significantly negative. To reduce potential interference from the later integration of the medical insurance system, we further restrict the sample to the 2012–2016 period. Column (5) continues to yield a negative and significant estimate. Finally, because housing construction year may partly capture housing quality, kitchen infrastructure, and the suitability of energy-using facilities, we additionally control for housing construction year. The results in Column (6) show that the baseline conclusion still holds. Overall, the core estimates remain stable across alternative specifications, suggesting that our findings are robust.

**Table 3:** Robustness checks.

Variables	(1) Lagged participation	(2) Village × year FE	(3) Add children control	(4) Excluding movers	(5) 2012–2016 only	(6) Add house build year control
	CleanEnergy					
InsRate		−0.058***	−0.044**	−0.045**	−0.059**	−0.056***
		(0.021)	(0.019)	(0.023)	(0.029)	(0.021)
L_InsRate	−0.041**					
	(0.021)					
Gender_head	−0.027**	−0.028**	−0.033***	−0.044***	−0.049***	−0.038***
	(0.013)	(0.012)	(0.011)	(0.013)	(0.015)	(0.0130)
Age_head	−0.004***	−0.004***	−0.004***	−0.003***	−0.004***	−0.003***
	(0.001)	(0.001)	(0.001)	(0.001)	(0.001)	(0.001)
Edu_head	0.007***	0.007***	0.007***	0.007***	0.008***	0.007***
	(0.002)	(0.002)	(0.002)	(0.002)	(0.002)	(0.002)
Marry_head	−0.072***	−0.062**	−0.062***	−0.070***	−0.045	−0.074***
	(0.025)	(0.024)	(0.023)	(0.027)	(0.032)	(0.0250)
Party_head	0.002	0.029	0.028	0.034	0.050*	0.004
	(0.028)	(0.025)	(0.023)	(0.028)	(0.027)	(0.029)
Familysize	−0.021***	−0.021***	−0.020***	−0.022***	−0.017***	−0.022***
	(0.004)	(0.004)	(0.004)	(0.004)	(0.005)	(0.004)
IncomeFam	0.050***	0.044***	0.043***	0.046***	0.040***	0.051***
	(0.006)	(0.006)	(0.006)	(0.006)	(0.007)	(0.006)
Asset	−0.001	−0.000	−0.001	−0.002	0.002	−0.001
	(0.001)	(0.001)	(0.001)	(0.001)	(0.002)	(0.001)
Subsidy_low	−0.001	0.003	0.002	0.003	−0.015	0.001
	(0.024)	(0.024)	(0.023)	(0.026)	(0.031)	(0.024)
ln_gdp	−0.117		−0.0460	−0.019	0.099	−0.112
	(0.088)		(0.072)	(0.079)	(0.118)	(0.091)
Children			0.012			
			(0.008)			
House build year						0.001**
						(0.001)
Constant	1.523*	0.392***	0.923	0.522	−0.651	−1.054
	(0.894)	(0.073)	(0.727)	(0.788)	(1.158)	(1.396)
Observations	7,319	8,754	8,777	6,588	4,381	6,830
*R*-squared	0.443	0.495	0.446	0.443	0.461	0.455
Village FE	Yes	Yes	Yes	Yes	Yes	Yes
Year FE	Yes	No	Yes	Yes	Yes	Yes
Village × year FE	No	Yes	No	No	No	No
Cluster	fid	fid	fid	fid	fid	fid

Cluster-robust standard errors, clustered at the household level (fid), are in parentheses.

### 4.3 Additional identification checks

Potential endogeneity in this study mainly stems from omitted variables and reverse causality. On the one hand, some unobserved household characteristics, such as health awareness or long-standing consumption habits, may simultaneously affect medical insurance participation and clean energy use. Although we alleviate potential endogeneity concerns by including a comprehensive set of control variables in the robustness checks, the possibility of bias arising from unobservable factors cannot be entirely ruled out. On the other hand, households’ energy choices may themselves be related to health conditions and economic circumstances, which can in turn influence insurance participation. To address these concerns, we further conduct four analyses.

#### 4.3.1 Lewbel method

As an additional identification check, we employ a two-stage least squares approach using an internally generated instrument. Following Lewbel (40), the instrument is constructed from higher-order moments of the residual obtained from the equation for the endogenous variable. In practice, we first obtain the residual, subtract its mean, and then use its cubic term as the internal instrument (41).

The estimation results from the Lewbel IV approach are reported in Table 4. In the first stage, the Lewbel IV is positively and significantly associated with InsRate at the 1% level, with a coefficient of 3.1381. The Kleibergen–Paap rk LM statistic is 321.532, with a *p*-value of 0.0000, which rejects the null hypothesis of underidentification. In addition, the Kleibergen–Paap rk Wald F statistic is 743.361, far exceeding the Stock–Yogo 10% maximal IV size critical value of 16.38, suggesting that weak identification is unlikely to be a concern. In the second stage, the coefficient on InsRate remains negative and statistically significant at the 5% level (−0.0537). These results indicate that the negative effect of medical insurance participation on household use clean energy as the primary cooking fuel persists after accounting for potential endogeneity concerns, thereby lending further support to the baseline findings.

**Table 4:** Results from the Lewbel IV estimates.

Variables	(1) First stage	(2) Second stage
	InsRate	CleanEnergy
Lewbel IV	3.138***	
	(0.115)	
InsRate		−0.054**
		(0.027)
Gender_head	0.031***	−0.034***
	(0.005)	(0.011)
Age_head	0.001***	−0.004***
	(0.000)	(0.001)
Edu_head	−0.002***	0.007***
	(0.001)	(0.002)
Marry_head	0.009	−0.061***
	(0.009)	(0.023)
Party_head	−0.005	0.030
	(0.010)	(0.023)
Familysize	−0.056***	−0.020***
	(0.002)	(0.004)
IncomeFam	−0.024***	0.043***
	(0.003)	(0.005)
Asset	0.003***	−0.001
	(0.000)	(0.001)
Subsidy_low	−0.023**	0.003
	(0.009)	(0.023)
ln_gdp	−0.074**	−0.054
	(0.029)	(0.072)
Observations	8,780	8,780
Kleibergen–Paap rk LM		321.532
Kleibergen–Paap rk LM *p*-value		0.0000
Kleibergen–Paap rk Wald *F*		743.361
*R*-squared	0.699	0.039
Village FE	Yes	Yes
Year FE	Yes	Yes
Cluster	fid	fid

Cluster-robust standard errors, clustered at the household level (fid), are reported in parentheses. Village and year fixed effects are included in all specifications. ***p < 0.01, **p < 0.05, *p < 0.10.

#### 4.3.2 Coefficient stability test

To further assess the sensitivity of the baseline results to omitted-variable bias, we conduct the coefficient-stability test proposed by Oster et al. (42, 43). Table 5 reports the result of the coefficient stability test. Following Oster (42), we set $R_{\max}$ to 1.3 times the R2 from the fully controlled specification, which yields $R_{\max}$ = 0.583. This value represents the hypothetical explanatory power that would be achieved if both observed and unobserved controls were included in the model. Under this assumption, the bias-adjusted coefficient remains negative at −0.028. The bias-adjusted coefficient refers to the estimated treatment effect after accounting for potential omitted-variable bias from unobservables. Its negative value suggests that the estimated effect is unlikely to be driven entirely by selection on unobserved factors.

**Table 5:** Results from the coefficient-stability test.

Rmax	Threshold	Estimated value
0.583	*β* * (Rmax, *δ*) ∈ [−0.100, −0.026]	*β* * (Rmax, *δ*) = −0.028
	|*δ*| > 1	*δ* = −1.850

Furthermore, we compute *δ*, which measures the relative strength of selection on unobservables versus observables required to eliminate the estimated effect. A larger absolute value of *δ* indicates greater robustness to omitted-variable bias, and values exceeding one suggest that unobserved selection would need to be stronger than observed selection to explain away the results. The estimated value of δ in our model is −1.850, with an absolute value greater than 1. Specifically, ∣δ∣ = 1.850 indicates that selection on unobservables would need to be about 1.85 times as strong as selection on observables to eliminate the estimated negative effect. Given that we have controlled for a comprehensive set of observable determinants of households’ clean energy use, unobserved factors would need to be substantially more influential than observed factors to overturn our results.

#### 4.3.3 Kinky least squares method

To further examine whether omitted variables correlated with insurance participation may affect our estimates, we employ the Kinky Least Squares (KLS) approach proposed by Kripfganz and Kiviet (44). Rather than relying on external instruments, KLS conducts inference by imposing assumptions on the admissible correlation, denoted by ρ , between the potentially endogenous regressor and the structural error term. This method has been widely used in existing literature (43, 45).

In our setting, we assume ρ is positive. This is because unobserved factors such as information access, cognitive ability, environmental awareness, village governance capacity, and local policy implementation may increase both household medical insurance participation and clean energy use. Since the baseline coefficient on InsRate is negative, such positive selection would tend to bias the estimated effect toward zero. Therefore, when stronger positive endogeneity is assumed, the KLS-adjusted coefficient is expected to become more negative.

Table 6 reports the KLS estimates under alternative assumed values of ρ . As ρ increases from 0.1 to 0.4, the absolute value of the coefficient on InsRate increases. This pattern reflects the adjustment for stronger positive endogeneity assumptions rather than necessarily indicating instability of the results. Importantly, across the full admissible range of ρ , the coefficient on InsRate remains negative and statistically significant. Thus, the KLS results suggest that the negative association between medical insurance participation and clean energy use is robust to plausible positive correlations between insurance participation and unobserved determinants of clean energy use.

**Table 6:** Results from kinky least squares estimation.

Variables	(1)	(2)	(3)	(4)
	CleanEnergy			
InsRate	−0.209***	−0.381***	−0.565***	−0.772***
	(0.017)	(0.019)	(0.023)	(0.029)
Gender_head	−0.029***	−0.025**	−0.020**	−0.014
	(0.009)	(0.010)	(0.010)	(0.010)
Age_head	−0.004***	−0.004***	−0.003***	−0.003***
	(0.000)	(0.000)	(0.000)	(0.000)
Edu_head	0.007***	0.006***	0.006***	0.005***
	(0.001)	(0.001)	(0.001)	(0.001)
Marry_head	−0.056***	−0.050***	−0.044***	−0.036**
	(0.016)	(0.016)	(0.016)	(0.017)
Party_head	0.031	0.031	0.032	0.033
	(0.020)	(0.021)	(0.021)	(0.023)
Familysize	−0.028***	−0.036***	−0.045***	−0.056***
	(0.003)	(0.003)	(0.003)	(0.003)
IncomeFam	0.041***	0.038***	0.035***	0.032***
	(0.004)	(0.004)	(0.005)	(0.005)
Asset	−0.000	0.000	0.001	0.001
	(0.001)	(0.001)	(0.001)	(0.001)
Subsidy_low	0.001	−0.002	−0.005	−0.009
	(0.017)	(0.017)	(0.018)	(0.019)
ln_gdp	−0.066	−0.078	−0.092	−0.108
	(0.066)	(0.067)	(0.069)	(0.072)
Village FE	Yes	Yes	Yes	Yes
Year FE	Yes	Yes	Yes	Yes
Observations	8,780	8,780	8,780	8,780
Assumed Correlation	0.1	0.2	0.3	0.4

Cluster-robust standard errors, clustered at the household level, are reported in parentheses. Village and year fixed effects are included in all specifications. ***p < 0.01, **p < 0.05, *p < 0.10.

#### 4.3.4 Instrumental variable methods

This study uses the hukou status of the household head as an instrumental variable for household medical insurance participation (InsRate) to further test the robustness of the baseline results. The validity of this instrument is grounded in the institutional features of China’s household registration system. Regarding relevance, hukou status plays a key role in shaping households’ access to and participation in public medical insurance programs. In particular, the New Rural Cooperative Medical Scheme (NRCMS) primarily covered rural residents in the early period, making rural hukou an important prerequisite for enrollment. Although hukou-based restrictions were gradually relaxed following the integration into the Urban–Rural Resident Basic Medical Insurance (URRBMI) after 2016, the enrollment patterns and renewal inertia formed under the earlier system have maintained a stable association between hukou status and insurance participation.

We construct the instrumental variable Hukou_head based on the hukou status of the household head. Specifically, Hukou_head is coded as 1 for urban hukou and 0 for rural hukou. Therefore, a larger value indicates urban hukou status and is expected to be associated with a lower participation rate in resident medical insurance.

Table 7 presents the IV estimation results. The first-stage results show that Hukou_head is significantly negatively associated with InsRate, indicating that households headed by individuals with rural hukou are more likely to participate in resident medical insurance. The Kleibergen–Paap rk LM statistic is 58.789, with a *p*-value of 0.0000, rejecting the null hypothesis of under-identification and indicating that the instrument is relevant. The Kleibergen–Paap rk Wald *F* statistic is 98.027, well above the conventional threshold of 10, indicating that weak-instrument concerns are unlikely to be severe. In the second stage, the coefficient on InsRate remains negative and statistically significant at −0.390, suggesting that medical insurance participation continues to reduce the likelihood that rural households use clean energy as their primary cooking fuel.

**Table 7:** Results from Instrumental variable analysis.

Variables	(1)	(2)
	First stage	2SLS
	InsRate	CleanEnergy
Hukou_head	−0.101***	
	(0.010)	
InsRate		−0.390***
		(0.126)
Gender_head	0.031***	−0.025**
	(0.007)	(0.012)
Age_head	0.001**	−0.004***
	(0.000)	(0.001)
Edu_head	−0.001	0.006***
	(0.001)	(0.002)
Marry_head	0.034**	−0.052**
	(0.014)	(0.023)
Party_head	0.021	0.040
	(0.015)	(0.024)
Familysize	−0.051***	−0.036***
	(0.002)	(0.007)
IncomeFam	−0.012***	0.039***
	(0.003)	(0.006)
Asset	0.003***	0.000
	(0.001)	(0.001)
Subsidy_low	−0.026*	−0.005
	(0.014)	(0.024)
ln_gdp	−0.054	−0.081
	(0.046)	(0.075)
Observations	8,657	8,657
Kleibergen–Paap rk LM		58.789
Kleibergen–Paap rk LM *p*-value		0.0000
Kleibergen–Paap rk Wald *F*		98.027
*R*-squared	0.318	−0.007
Village FE	Yes	Yes
Year FE	Yes	Yes
Cluster	fid	fid

Cluster-robust standard errors, clustered at the household level (fid), are reported in parentheses. Village and year fixed effects are included in all specifications. ***p < 0.01, **p < 0.05, *p < 0.10.

Nevertheless, hukou status may affect household clean energy use through channels other than medical insurance, which poses a potential challenge to the exogeneity assumption of our instrumental variable. To mitigate this concern, we further examine whether the hukou status of the household head significantly predicts several observable channels through which hukou status could directly influence household clean energy use, including household assets, housing construction year, and village-level medical access barrier. These variables capture important differences in household economic capacity, housing conditions, and barriers to local medical access, respectively, and thus represent potential pathways through which the instrumental variable might affect the use of clean cooking energy. The results, reported in Table 8, show that hukou status does not significantly predict these observable factors. This suggests that hukou status is less likely to affect household clean energy use directly through these channels. Although the exclusion restriction cannot be directly tested, these results provide indirect support for the plausibility of the instrumental variable.

**Table 8:** Supplementary evidence on the exclusion restriction of the hukou-based instrumental variable.

Variables	(1)	(2)	(3)
	Asset	House build year	Medical access barrier
Hukou_head	0.142	−0.752	−0.017
	(0.092)	(0.501)	(0.026)
Gender_head	0.158	−0.902	0.030
	(0.118)	(0.568)	(0.026)
Age_head	−0.002	−0.202***	0.004***
	(0.006)	(0.028)	(0.001)
Edu_head	0.039**	−0.025	−0.011**
	(0.017)	(0.077)	(0.005)
Marry_head	0.728***	0.330	−0.074
	(0.230)	(1.135)	(0.048)
Party_head	0.087	0.945	−0.013
	(0.217)	(0.851)	(0.047)
Familysize	−0.002	0.082	0.009
	(0.034)	(0.162)	(0.009)
IncomeFam	0.465***	0.250***	−0.030***
	(0.067)	(0.093)	(0.011)
Asset		−0.231	−0.002
		(1.349)	(0.002)
Subsidy_low	−0.319	5.689	0.075
	(0.292)	(4.711)	(0.057)
ln_gdp	1.108*	0.761***	0.064**
	(0.644)	(0.256)	(0.025)
Observations	8,657	2,803	7,533
*R*-squared	0.140	0.342	0.498
Village FE	Yes	Yes	Yes
Year FE	Yes	Yes	Yes
Cluster	fid	fid	fid

Cluster-robust standard errors, clustered at the household level (fid), are reported in parentheses. Village and year fixed effects are included in all specifications. ***p < 0.01, **p < 0.05, *p < 0.10.

### 4.4 Mechanism analysis

The baseline results and a series of robustness checks consistently show that participation in medical insurance significantly reduces rural households’ use of clean energy. However, the underlying mechanism remains unclear. According to the theoretical framework of this study, both clean energy and medical care can be viewed as health investments that contribute to the accumulation of health capital. By lowering the cost of medical care and improving access to healthcare services, medical insurance is expected to increase households’ utilization of medical care and related expenditures. To examine this mechanism, we conduct a set of empirical tests focusing on healthcare utilization and medical expenditures.

Table 9 reports the results of the mechanism analysis. The results provide supportive evidence for the proposed mechanism. First, column (1) shows that the coefficient on InsRate is 0.036 and statistically significant at the 5% level, indicating that participation in medical insurance significantly increases the likelihood of hospitalization. This suggests that insurance coverage promotes the use of medical care services. Second, columns (2)–(4) show that InsRate has positive and statistically significant effects on OOPMedical (0.660), HospCost (0.312), and MedicalExp (0.438), indicating that insurance participation not only increases medical care utilization but also raises out-of-pocket medical expenditures, inpatient expenditures, and total medical spending. Finally, column (5) shows that the coefficient on InsRate for MedShare is 0.015 and statistically significant at the 5% level, suggesting that the share of medical expenditure in total household spending increases following insurance participation.

**Table 9:** Results from mechanism analysis.

Variables	(1)	(2)	(3)	(4)	(5)
	Hospitalized	OOPMedical	HospCost	MedicalExp	MedShare
InsRate	0.036**	0.660***	0.312**	0.438***	0.015**
	(0.015)	(0.147)	(0.124)	(0.127)	(0.007)
Gender_head	−0.011	−0.846***	−0.054	−0.156**	−0.008*
	(0.009)	(0.089)	(0.078)	(0.078)	(0.004)
Age_head	0.002***	0.031***	0.020***	0.018***	0.002***
	(0.000)	(0.004)	(0.003)	(0.004)	(0.000)
Edu_head	−0.002*	−0.005	−0.017	0.020*	−0.001
	(0.001)	(0.012)	(0.011)	(0.011)	(0.001)
Marry_head	0.007	0.140	0.094	0.497***	0.011
	(0.018)	(0.163)	(0.156)	(0.151)	(0.008)
Party_head	0.001	−0.114	0.009	−0.063	−0.008
	(0.016)	(0.146)	(0.141)	(0.134)	(0.008)
Familysize	−0.002	−0.039	−0.020	0.176***	−0.000
	(0.003)	(0.028)	(0.024)	(0.024)	(0.001)
IncomeFam	−0.002	0.004	−0.019	0.044	−0.016***
	(0.004)	(0.038)	(0.033)	(0.034)	(0.002)
Asset	−0.002*	−0.023***	−0.015*	−0.020***	−0.002***
	(0.001)	(0.009)	(0.009)	(0.007)	(0.000)
Subsidy_low	0.014	0.163	0.091	−0.149	−0.008
	(0.018)	(0.165)	(0.153)	(0.128)	(0.007)
ln_gdp	−0.033	−0.052	−0.385	1.146**	0.028
	(0.051)	(0.478)	(0.426)	(0.474)	(0.024)
Observations	8,780	8,780	8,780	8,780	8,780
*R*-squared	0.084	0.316	0.083	0.137	0.137
Village FE	Yes	Yes	Yes	Yes	Yes
Year FE	Yes	Yes	Yes	Yes	Yes
Cluster	fid	fid	fid	fid	fid

Cluster-robust standard errors, clustered at the household level (fid), are reported in parentheses. Village and year fixed effects are included in all specifications. ***p < 0.01, **p < 0.05, *p < 0.10.

Our mechanism analysis provides suggestive evidence in support of the proposed hypotheses. On the one hand, the findings are consistent with the theory of ex ante moral hazard. This theory argues that health insurance, by reducing the financial consequences of illness and lowering the effective price of medical care, may weaken individuals’ or households’ incentives to undertake self-protective health investments at the margin (18). Empirical evidence also shows that health insurance increases healthcare utilization, particularly inpatient care utilization (19), and may reduce certain preventive health behaviors or self-protective investments (22, 46).

On the other hand, clean cooking energy use can be regarded as an important form of household health investment, as reliance on solid cooking fuels may increase household medical expenditures (47), while the adoption of cleaner energy sources can help reduce medical spending (48). However, using clean cooking energy requires continuous fuel payments and certain equipment-related investments, making it highly dependent on household affordability and energy budget constraints (49, 50). Medical expenditures, especially out-of-pocket and catastrophic health expenditures, can reshape household consumption patterns and crowd out other non-medical expenditures (51–53). Therefore, when medical insurance increases the probability of hospitalization and raises household medical spending, households may rely more on ex post medical care to cope with health risks while reducing their allocation to ex ante health investments, such as clean cooking energy use, under budget constraints. In this sense, increased healthcare utilization and medical expenditure may inhibit household clean cooking energy use through both risk-substitution and budget-crowding-out channels.

### 4.5 Heterogeneity analysis

To further examine whether the effect of medical insurance participation on clean energy use as the primary cooking fuel differs across household types, this study introduces interaction terms into the baseline model to test whether household characteristics significantly moderate the relationship between medical insurance participation and clean energy use. We examine heterogeneity along three dimensions: the old-age dependency ratio, household assets, and medical access barrier.

Specifically, the old-age dependency ratio is used to measure the care burden associated with older household members. Households with an old-age dependency ratio above the sample mean are defined as households with a high old-age dependency ratio and assigned a value of 1, and 0 otherwise. Household assets are used to capture the household’s economic foundation and budget constraints, while medical access barrier is measured by the time required for the household to reach the nearest medical facility. Based on these definitions, we construct interaction terms between medical insurance participation and high old-age dependency ratio, household assets, and medical access barrier, respectively.

Table 10 reports the results of the heterogeneity analysis. Column (1) shows that the interaction term between InsRate and high old-age dependency ratio is −0.069 and statistically significant at the 10% level. This result suggests that, compared with households with a lower old-age dependency ratio, the negative association between medical insurance participation and clean energy use is significantly stronger among households with a high old-age dependency ratio. These households usually face a heavier care burden associated with older household members and greater potential medical needs. Therefore, when medical insurance improves access to healthcare services, they may allocate more resources to healthcare utilization and medical expenditures, thereby reducing their investment capacity for clean energy use.

**Table 10:** Heterogeneity analysis.

Variables	(1)	(2)	(3)
	Old-age dependency ratio	Household assets	Medical access barrier
InsRate	−0.012	−0.365**	0.042
	(0.023)	(0.158)	(0.055)
High old-age dependency ratio	−0.019		
	(0.025)		
InsRate * high old-age dependency ratio	−0.069*		
	(0.036)		
Household assets		0.003	
		(0.008)	
InsRate * household assets		0.026**	
		(0.012)	
Medical access barrier			0.001
			(0.017)
InsRate * medical access barrier			−0.042*
			(0.024)
Gender_head	−0.034***	−0.036***	−0.040***
	(0.011)	(0.011)	(0.012)
Age_head	−0.002***	−0.003***	−0.003***
	(0.001)	(0.001)	(0.001)
Edu_head	0.006***	0.006***	0.006***
	(0.002)	(0.002)	(0.002)
Marry_head	−0.069***	−0.068***	−0.067***
	(0.023)	(0.023)	(0.024)
Party_head	0.032	0.028	0.036
	(0.023)	(0.023)	(0.025)
Familysize	−0.020***	−0.020***	−0.020***
	(0.004)	(0.004)	(0.004)
IncomeFam	0.033***	0.037***	0.040***
	(0.005)	(0.005)	(0.006)
Asset	0.009	0.008	0.011
	(0.023)	(0.023)	(0.025)
Subsidy_low	−0.068	−0.065	−0.033
	(0.072)	(0.072)	(0.075)
ln_gdp	−0.034***	−0.036***	−0.040***
	(0.011)	(0.011)	(0.012)
Observations	8,780	8,780	7,626
*R*-squared	0.449	0.447	0.440
Village FE	Yes	Yes	Yes
Year FE	Yes	Yes	Yes
Cluster	fid	fid	fid

Robust standard errors are clustered at the household level and reported in parentheses. Fixed effects are indicated in the table. ***, **, and * denote statistical significance at the 1%, 5%, and 10% levels, respectively.

Column (2) shows that the coefficient of InsRate is significantly negative, while the interaction term between InsRate and household assets is 0.026 and statistically significant at the 5% level. This result indicates that, compared with households with higher asset holdings, the negative association between medical insurance participation and clean energy use is more pronounced among low-asset households. Since low-asset households generally face tighter budget constraints, increased healthcare utilization and medical expenditures may more easily crowd out their investment in clean cooking fuels, energy equipment, and related household facilities.

Column (3) shows that the interaction term between InsRate and medical access barrier is −0.042 and statistically significant at the 10% level. This result suggests that, compared with households with low medical access barrier, the negative association between medical insurance participation and clean energy use is stronger among households with greater barriers to medical access. Greater barriers to medical access implies higher time costs of obtaining healthcare services and may also reflect stronger healthcare constraints and greater uncertainty about future medical needs. Under such conditions, medical insurance participation may further strengthen households’ reliance on curative health inputs and affect their allocation of resources between medical expenditures and clean energy use.

Overall, the heterogeneity results suggest that the negative association between medical insurance participation and clean energy use is mainly concentrated among households with a high old-age dependency ratio, relatively limited household assets, and greater barriers to medical access. These households tend to face greater potential medical needs, tighter budget constraints, or poorer access to healthcare services. After participating in medical insurance, they may allocate more limited resources to healthcare utilization and medical expenditures, thereby weakening their investment capacity for clean energy use. These results further indicate that the relationship between medical insurance participation and clean energy use is not uniform across households, but is moderated by household demographic structure, asset holdings, and medical accessibility.

## 5 Conclusion and discussion

### 5.1 Conclusion

This study uses data from the China Family Panel Studies (CFPS) to examine the effect of rural household medical insurance participation on clean energy use. Based on a two-way fixed effects model, instrumental variable estimation, and a series of robustness checks, we find that a higher household medical insurance participation rate significantly reduces the likelihood that rural households use clean cooking energy as the primary cooking fuel. The mechanism analysis shows that medical insurance significantly increases the likelihood of hospitalization, while also raising out-of-pocket medical expenses, hospitalization expenses, total household medical expenditure, and the share of medical expenditure in total household spending. These results suggest that medical insurance increases household utilization of medical services and increases medical expenditures. Therefore, from the perspective of substitution among health investments, households may reduce their investment in clean energy. The heterogeneity analysis further indicates that the negative effect of medical insurance on clean energy use is more pronounced among households with aging family structures, lower asset holdings, and greater barriers to medical access.

### 5.2 Discussion

This study shows that, while medical insurance improves access to medical care, it may also crowd out clean energy use through a health investment substitution effect, thereby reducing rural households’ use of clean energy as the primary cooking fuel. This finding has important policy implications. Existing policies often treat medical insurance expansion and clean energy promotion as independent policy instruments. However, our results suggest that these two forms of health investment may act as substitutes in the production of health capital. By lowering the cost of medical care and encouraging medical care utilization, medical insurance may reduce households’ incentives to invest in preventive health measures such as clean energy. This may weaken the preventive health benefits associated with clean energy use and potentially increase future medical care demand. Therefore, policymakers should account for the indirect behavioral effects of medical insurance on other health-related investments.

In light of these findings, policy design should move toward a more integrated approach that coordinates different forms of health investment. Simply increasing medical insurance subsidies may alleviate the financial burden of medical care but could also reinforce reliance on medical services and intensify the substitution effect. Instead, governments could consider linking medical insurance expansion with clean energy promotion through targeted and differentiated subsidy schemes.

Such a policy is feasible in the rural Chinese context. Village committees usually have detailed knowledge of local households’ energy use, income status, and living conditions, and they already play an important role in implementing public programs at the grassroots level. By using village-level registration systems and digital household records, local governments can identify households that still rely on traditional solid fuels at relatively low administrative cost. Eligibility verification could be combined with existing administrative procedures for medical insurance enrollment, poverty assistance, or rural household registration, thereby avoiding the need to establish a separate policy implementation system.

For example, households that continue to rely on firewood or coal could receive a one-time clean energy voucher, equipment subsidy, or in-kind support for adopting electric stoves, gas stoves, or other clean cooking technologies when enrolling in medical insurance programs. Compared with continuous cash transfers, equipment-based subsidies may be more administratively manageable because clean energy devices have a relatively long service life and can generate health benefits over several years. This design also helps reduce the fiscal burden by concentrating support on the initial adoption cost rather than providing permanent operating subsidies.

Potential adverse selection should also be considered. Households may strategically report continued use of traditional fuels in order to qualify for subsidies. To reduce this risk, eligibility could be verified through village committee confirmation, household energy-use records, purchase receipts, or periodic spot checks. Subsidies could also be provided in the form of vouchers or direct provision of clean energy equipment rather than unrestricted cash transfers, which would help ensure that public funds are used for clean energy use. In addition, targeting can prioritize households with higher health risks or stronger budget constraints, such as low-asset households, households with a high old-age dependency ratio, or households facing greater barriers to medical access.

Although such measures may increase fiscal expenditures in the short run, they may reduce pollution-related health risks and associated medical costs over time. Therefore, linking medical insurance with clean energy support can help mitigate the crowding-out effect identified in this study and achieve both public health improvement and clean energy use objectives. Overall, policy interventions should recognize the interactions among different health investments and aim to align medical care and environmental policies to promote both health improvement and sustainable energy use.

### 5.3 Limitations

This study has several limitations. First, the dependent variable is based on households’ reported primary cooking fuel and may not fully capture fuel stacking or the intensity of clean energy use. Since our measure is a binary indicator based on the household’s primary cooking fuel in each survey year, it cannot capture gradual changes in households’ energy structure. Future studies could use more detailed data on fuel expenditure, consumption quantities, or usage frequency to examine the continuous adjustment of household energy portfolios.

Second, due to data limitations, we cannot directly observe detailed local clean energy subsidies or household-level energy prices. This prevents us from directly examining whether energy subsidies can moderate the negative effect of medical insurance on clean energy use. Therefore, our results provide suggestive rather than definitive evidence for the potential role of clean energy subsidies in mitigating the crowding-out effect of medical insurance. Future studies with more detailed policy and price data could further examine whether targeted energy subsidies can effectively promote clean energy adoption among insured rural households.

Third, China’s rural medical insurance system has achieved broad coverage and relatively high reimbursement rates, whereas health insurance schemes in other developing countries may provide lower reimbursement rates and weaker financial protection. As a result, the extent to which medical insurance increases healthcare utilization and crowds out clean energy investment may vary across institutional contexts. Future research could examine whether similar health-investment substitution effects exist in countries with different health insurance systems, reimbursement levels, and rural energy infrastructures.

## Data Availability

Publicly available datasets were analyzed in this study. This data can be found at: https://cfpsdata.pku.edu.cn/#/home.
